# Automatic cortical surface parcellation in the fetal brain using attention-gated spherical U-net

**DOI:** 10.3389/fnins.2024.1410936

**Published:** 2024-05-30

**Authors:** Sungmin You, Anette De Leon Barba, Valeria Cruz Tamayo, Hyuk Jin Yun, Edward Yang, P. Ellen Grant, Kiho Im

**Affiliations:** ^1^Fetal Neonatal Neuroimaging and Developmental Science Center, Boston Children’s Hospital, Harvard Medical School, Boston, MA, United States; ^2^Division of Newborn Medicine, Boston Children’s Hospital, Harvard Medical School, Boston, MA, United States; ^3^Department of Pediatrics, Harvard Medical School, Boston, MA, United States; ^4^Department of Radiology, Boston Children’s Hospital, Harvard Medical School, Boston, MA, United States

**Keywords:** fetal MRI, brain MRI, cortical surface parcellation, deep learning, spherical U-net, attention mechanism

## Abstract

Cortical surface parcellation for fetal brains is essential for the understanding of neurodevelopmental trajectories during gestations with regional analyses of brain structures and functions. This study proposes the attention-gated spherical U-net, a novel deep-learning model designed for automatic cortical surface parcellation of the fetal brain. We trained and validated the model using MRIs from 55 typically developing fetuses [gestational weeks: 32.9 ± 3.3 (mean ± SD), 27.4–38.7]. The proposed model was compared with the surface registration-based method, SPHARM-net, and the original spherical U-net. Our model demonstrated significantly higher accuracy in parcellation performance compared to previous methods, achieving an overall Dice coefficient of 0.899 ± 0.020. It also showed the lowest error in terms of the median boundary distance, 2.47 ± 1.322 (mm), and mean absolute percent error in surface area measurement, 10.40 ± 2.64 (%). In this study, we showed the efficacy of the attention gates in capturing the subtle but important information in fetal cortical surface parcellation. Our precise automatic parcellation model could increase sensitivity in detecting regional cortical anomalies and lead to the potential for early detection of neurodevelopmental disorders in fetuses.

## 1 Introduction

Cortical surface parcellation of the human brain refers to the process of dividing the cerebral cortex into distinct regions based on various criteria, including anatomical landmarks, functional properties, connectivity patterns, or developmental trajectories (Fischl et al., 2004; Xia et al., 2019; McGrath et al., 2022). Anatomical cortical parcellations based on sulcal/gyral folding patterns using magnetic resonance imaging (MRI) have been widely used for various region-based cortical structural, functional, and network analyses. Regional analysis via cortical surface parcellation is even important for fetal brains since regional variations in brain structures affected by genetic and environmental factors and neurodevelopmental disorders already occur during this period (Rajagopalan et al., 2011; Vasung et al., 2016, 2020, 2021; Andescavage et al., 2017; Ortinau et al., 2019).

As manual cortical parcellation is labor-intensive, reliant on expert knowledge, and time-consuming, several automated methods have been proposed for sulcal/gyral parcellations on adult and infant cortical surfaces from MRI (Fischl et al., 2004; Lyttelton et al., 2007; Destrieux et al., 2010; Yeo et al., 2010; Li and Shen, 2011; Auzias et al., 2016; Gopinath et al., 2019; Parvathaneni et al., 2019; Zhao et al., 2019, 2021; Cheng et al., 2020; Hao et al., 2020). One prevalent approach to automatic parcellation involves surface registration, where single or probabilistic label maps defined on a reference surface are transferred to the target individual surface following registration (Fischl et al., 2004; Lyttelton et al., 2007; Destrieux et al., 2010; Yeo et al., 2010; Hazlett et al., 2017; Wu et al., 2019; Yun et al., 2019). However, the accuracy of traditional techniques for cortical surface parcellation is heavily dependent on the precise registration of the cortical surface, which establishes correspondences between the atlas and the subject.

With the advent and success of deep learning in computer vision (Deng et al., 2009), deep learning techniques were introduced for automatic cortical parcellation (Zhao et al., 2022). One approach was transforming the parcellation task into a common image classification task by making 2D patches from the cortical surface. Wu et al. (2018) directly applied a 2D convolutional neural network (CNN) to the cortical shape features, mean curvature, sulcal depth, and average convexity, to learn the nonlinear mapping to parcellation labels by projecting surface patches into tangent spaces to create regular 2D image patches and subsequently classifying those patches. However, the patch-wise approach inherently suffers from limitations such as the trade-off between spatial contextual information and localization during the patching process and redundant computations due to overlapping patches.

In order to overcome those issues, spherical CNNs (Jiang et al., 2019; Zhao et al., 2019) were proposed, which use the ring convolutional filters via spatial re-tessellation of the spherical surface onto the standard icosahedron. By the re-tessellation onto the icosahedron, the spherical surface could be transformed into a consistent structure with uniform-sampled vertices, which enabled the learning of feature maps on the spherical space with hierarchical CNN architectures (Zhao et al., 2021). As the U-net and its variations have shown state-of-the-art performances in medical segmentation fields (Ronneberger et al., 2015), Zhao et al. (2019) proposed a spherical U-net by replacing 2D convolution and pooling operators in the U-net with spherical ring-convolution and pooling. Another recent study proposed a deep learning model named SPHARM-net for cortical surface parcellation (Ha and Lyu, 2022), which introduced spherical harmonics-based convolution filters that can encode all the spectral components without the full harmonic expansion to capture geometric details. They applied the spherical harmonic convolution to the spherical U-net structure. Those studies based on spherical U-net structure showed improved performance for cortical surface parcellation compared to surface registration-based approaches in both adult and infant brains (Hao et al., 2020; Zhao et al., 2021; Ha and Lyu, 2022).

To the best of our knowledge, there have been no studies reporting automatic cortical parcellation for the fetal brain. Unlike infant or adult brains, fetal brains have small sizes and weights, smooth surfaces with limited gyrification, and immature regional structures (Habas et al., 2012; Dubois et al., 2014; Dubois and Dehaene-Lambertz, 2015). In addition, fetal brains have variations in relative position and size of cortical folds along with gestational age (GA). Since automatic cortical parcellation learns the mapping between cortical folding features and regional labels, cortical region definition and parcellation are challenging for fetal brains with immature cortical folding. Due to those reasons, previous methods developed for postnatal brains might not be robust enough to handle the unique anatomy of the fetal brain surface.

A deep learning model with the attention mechanism, inspired by human cognitive processes, allows it to focus selectively on specific parts of the input data, emphasizing regions of interest while downplaying less relevant areas (Vaswani et al., 2017; Wang et al., 2017; Oktay et al., 2018). This characteristic may be beneficial to address the challenges in the cortical surface parcellation of the fetal brain. First, it may help the model find and focus on subtle but important features from incomplete cortical folding maps of the fetal brain (Schlemper et al., 2019). Secondly, fetal brains have wide temporal variations in their folding characteristics under the neurodevelopment process. Therefore, the parcellation model should adaptively adjust the model’s internal focus on input feature maps according to GA (Jetley et al., 2018). Third, it can distinguish between genuine cortical features and imaging artifacts ensuring that the latter do not adversely affect the parcellation process (Liu et al., 2022). Lastly, it may make more informed decisions about the boundaries and classifications of cortical areas through the extension of receptive fields to consider the relationships between different brain regions widely (Zhang et al., 2018).

In this study, we propose an attention-gated spherical U-net by applying the attention mechanism to the spherical U-net for fetal cortical surface parcellation. The utilization of the attention mechanisms in the cortical surface parcellation model may promise a breakthrough to address the challenges posed by the unique fetal brain structure, allowing the model to focus adaptively on relevant features and contexts, which can significantly enhance the accuracy, robustness, and generalizability of fetal brain parcellation models.

## 2 Methods

### 2.1 Subjects and MR image acquisition

We collected MRIs from 55 typically developing (TD) fetuses [GA (mean ± SD, range): 32.9 ± 3.3 weeks, 27.4–38.7 weeks; sex (*n*, male/female/unknown): 23/12/20] for this study from prior prospective recruitment studies or clinical fetal MRIs that were performed to screen for fetal brain abnormalities but were clinically interpreted as normal by two board-certified radiologists. To construct a confirmed parcellation dataset, we included fetuses with successful cortical surface reconstruction and over 27 gestational weeks when gyral and sulcal folding starts visibly forming. This study was reviewed and approved by the Institutional Review Board at Boston Children’s Hospital. We acquired fetal brain MRIs on a Siemens 3 T Skyra scanner using a T2-weighted Half-Fourier Acquisition Single-Shot Turbo Spin-Echo (HASTE) sequence with the following parameters: in-plane resolution of 1 mm, field of view (FOV) of 256 mm × 256 mm, time repetition of 1.6 s, time echo of 120 ms, and slice thickness of 2–4 mm. After localization of fetal brains, multiplanar HASTE stacks were acquired at least three times in different orthogonal orientations to reconstruct reliable 3D motion-corrected volume of the fetal brain.

### 2.2 MRI processing

We used our fetal MRI processing pipeline (Im et al., 2017; Yun et al., 2020, 2021) to reconstruct the cortical surfaces, which consists of the brain extraction (Hong et al., 2021), the isotropic high-resolution volume reconstruction algorithm via a slice-to-volume registration (Kuklisova-Murgasova et al., 2012), and deep learning-based cortical plate (CP) segmentation (Hong et al., 2020). For the brain extraction, we used our in-house fetal brain extraction model based on a 2D U-net structure, which had been trained with 291 MRI stacks from 65 typical developing (TD) fetuses.^1^ After brain extraction, we corrected intensity inhomogeneity via N4 bias field correction (Tustison et al., 2010) and created a motion-corrected 3D volume with 0.75 mm isotropic resolution using a slice-to-volume super-resolution technique (Kuklisova-Murgasova et al., 2012). Then, we applied our automatic CP segmentation algorithm that had been developed for the fetal brain (Hong et al., 2020; https://github.com/jwhong1125/fetal_CP_segmentation). The CP segmentation algorithm is based on 2D U-net models trained separately for the sagittal, coronal, and axial planes, which had been trained with 52 TD fetuses. It includes multi-view aggregation and test-time augmentation for precise CP segmentation onto the 3D volumes.

After CP segmentation on 3D volumes, we extracted 3D inner CP surfaces using matching-cube algorithms from the CIVET,^2^ which generates tessellated triangular meshes for the boundary between the CP and its inner region. The algorithm tessellates a surface by collapsing an outer ellipsoid mesh enclosing the inner CP volumes and resampling it to the standard mech format with 81,920 triangles and 40,962 vertices (Liu et al., 2021). Lastly, we geometrically smoothed the resampled surface with the Taubin smoothing approach (Taubin, 1995) to obtain a natural shape of the surface without shrinking and voxelated patterns. In order to get brain surfaces on the standard mesh structure, we flipped the right hemisphere to the left, resampled onto the standard mesh, and re-flipped to the right. In this manner, both hemispheres share the same vertex indices and the neighborhood definition following the standard mesh structure, which enables the usage of both hemispheres together in the same model. After the resampling of individual cortical surfaces onto the standard mesh, both left and right surface models have the same vertex indices and neighborhoods. Therefore, both hemispheres were used for the training and evaluation of the parcellation models together.

**Figure 1:**
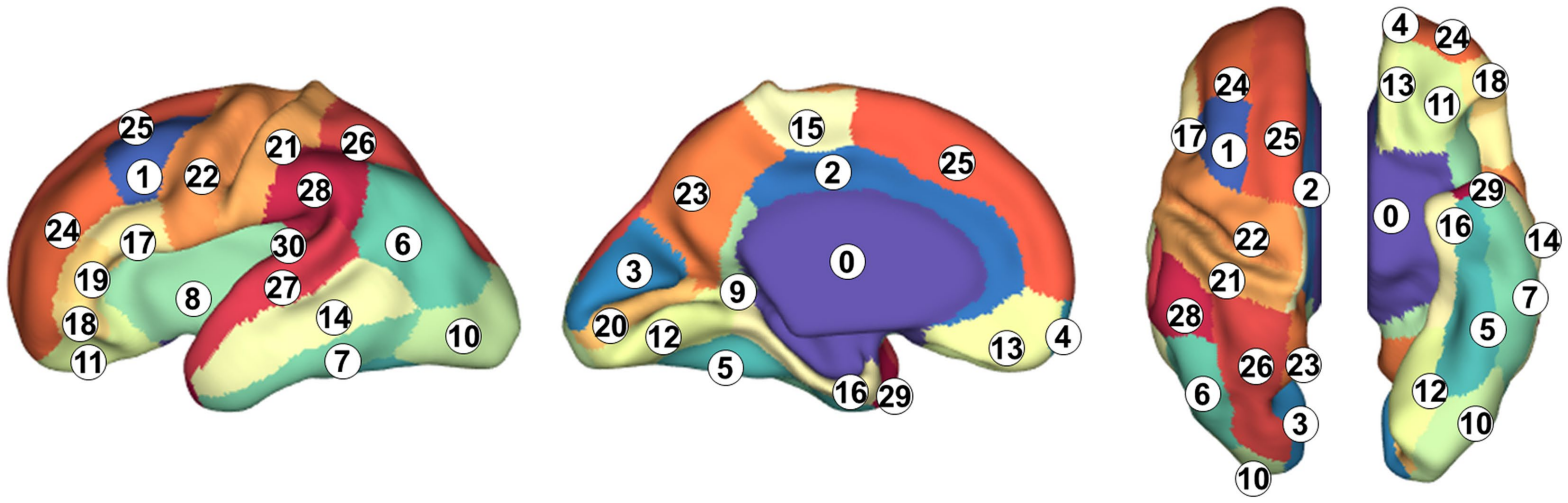
Cortical parcellation and anatomical labels. 0: background, 1: caudal middle frontal gyrus, 2: cingulate cortex, 3: cuneus, 4: frontal pole, 5: fusiform gyrus, 6: inferior parietal gyrus, 7: inferior temporal gyrus, 8: insula, 9: isthmus of the cingulate cortex, 10: lateral occipital cortex, 11: lateral orbital frontal cortex, 12: lingual gyrus, 13: medial orbital frontal cortex, 14: middle temporal gyrus, 15: parecentral lobule, 16: parahippocampal gyrus, 17: pars opercularis, 18: pars orbitalis, 19: pars triangularis, 20: pericalcarine cortex, 21: postcentral gyrus, 22: precentral gyrus, 23: precuneus cortex, 24: rostal middle frontal gyrus, 25: superior frontal gyrus, 26: superior parietal gyrus, 27: superior temporal gyrus, 28: supramarginal gyrus, 29: temporal pole, and 30: transverse temporal gyrus.

We computed three folding feature maps, mean curvature, average convexity, and adaptive distance transform-based sulcal depth (Yun et al., 2013) as inputs for surface parcellation. The mean curvature measures the cortical folding in a fine view, the average convexity measures the cortical folding in a coarse view, and our adaptive distance transform-based sulcal depth measures the cortical folding by combining both the coarse and fine views. For the parcellation label, we manually parcellated individual cortical surfaces following the Freesurfer Desikan parcellation protocol that has been extensively used as a standard in neuroimaging studies (Desikan et al., 2006). The original Desikan parcellation map consists of 34 cortical regions in each hemisphere. However, the secondary and tertiary sulci are not fully developed in the fetal brain, so it is not feasible to parcellate subdivisions of the gyrus. We simplified the original map and defined 30 cortical regions in each hemisphere (Figure 1).

### 2.3 Network architecture

**Figure 2:**
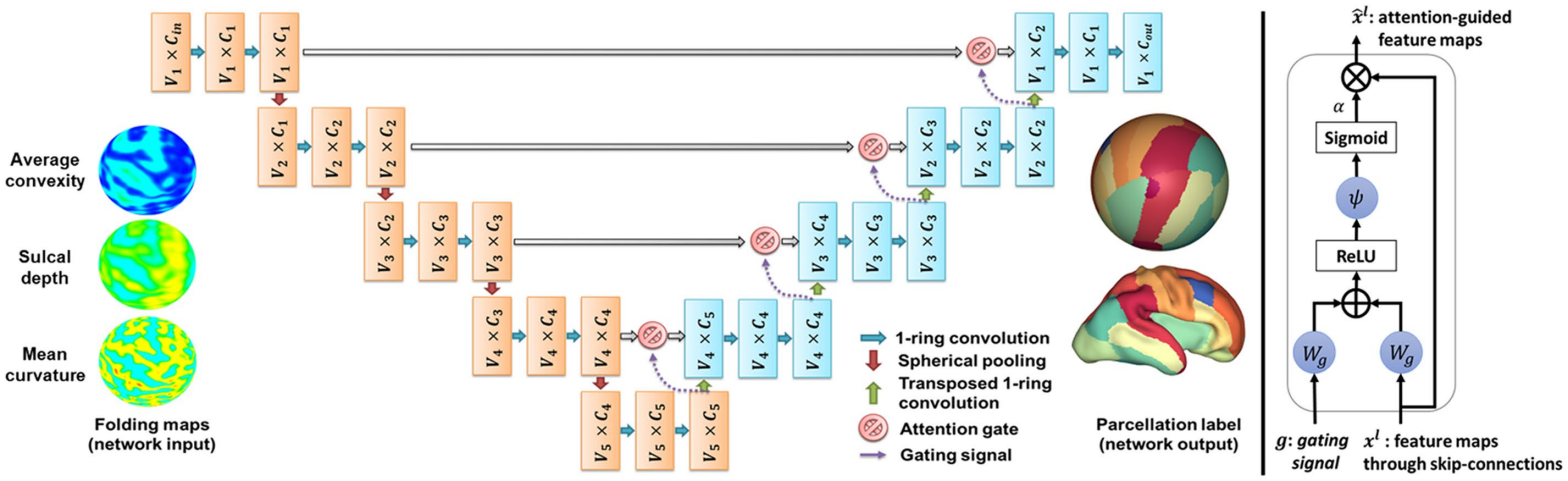
Schematic of the proposed cortical automatic parcellation mode with attention-gated spherical U-net. Left: The network receives cortical features mapped on the icosahedron with 40,962 vertices. In the encoder layers, ring convolutions and spherical pooling layers project feature maps onto icosahedrons with 10,242, 2,562, 642, and 162 vertices. Meanwhile, the number of channels at each layer is increased from 3 $\left(C_{\text{in}}\right)$ to 32, 64, 128, 256, and 512, respectively. In the decoding layers, transposed one-ring convolution layers upsample and project feature maps into the icosahedron with 40,962 vertices eventually. Right: The attention gate receives feature maps through skip connections and corresponding gating signals as inputs. The input feature maps are weighted by attention coefficients $\left(\alpha \right)$ computed within them.

In this study, we implemented an attention-gated spherical U-net by modifying the attention module and applying it to the original spherical U-net as backbone architecture (Figure 2). The spherical U-net architecture has an encoder path and a decoder path each with five up/down-sampling steps. Each path consists of repeated layers of convolution, batch normalization, and leaky rectified linear units like the original U-net. However, there are several differences from the original U-net in handling spherical structures as input and output. The general 2D convolution layers are replaced with ring convolutions. The ring convolutions are designed to perform convolution operations on the mesh structures (Zhao et al., 2021). Likewise, the up-convolution and max pooling are replaced with surface-transposed convolutions and surface mean pooling. The final layer will be a vertex-wise filter to map the feature vector to the output surface shape.

### 2.4 Attention gates for spherical U-net

Attention mechanisms can potentially enhance the network’s ability to focus on relevant features while ignoring irrelevant ones, thereby improving the accuracy and robustness of cortical surface parcellation (Oktay et al., 2018). The coefficient of attention, $\alpha_{i}\in \left[0,1\right],$ identify salient regions and prune feature response to preserve only the activations relevant to the specific task of the network which is the parcellation in this study. The output of the attention gate is the vertex-wise multiplication of input feature maps and attention coefficients, ${\hat{x}}_{i,c}^{l}=x_{i,c}^{l}\cdot \alpha_{i}^{l}$ (Figure 2). We used the multi-dimensional attention coefficients (Oktay et al., 2018) that enable each attention gate to learn to focus on multiple target regions of interest on each level of the icosahedron. The gating vector, $g_{i}\in {\mathbb{R}}^{F_{g}}$, is defined as multi-dimensional attention coefficients for each vertex i on the input icosahedron, where $F_{g}$ corresponds to the number of feature maps in layer l. The gating vector contains contextual information to determine regions to be focused on lower-level feature maps. We used the additive attention (Bahdanau et al., 2014) to get the gating vector for spherical feature maps on each level of the icosahedron.


$$q_{att}^{l}=\psi^{T}\left(\text{ReLU}\left(W_{x}^{T}x_{i}^{l}+W_{ga}^{T}g_{i}+b_{g}\right)\right)+b_{\psi }$$

$$\alpha_{i}^{l}=\text{sigmoid}\left(q_{att}^{l}\left(x_{i}^{l},\;g_{I};\Theta_{att}\right)\right)$$

The attention gate is defined as a set of parameters $\Theta_{att}$ containing: three linear transformations with one-ring convolution $W_{x}\in {\mathbb{R}}^{F_{l}\times F_{int}},W_{g}\in {\mathbb{R}}^{F_{g}\times F_{int}},\psi \in {\mathbb{R}}^{F_{l}\times 1}$ and bias terms $b_{\psi }\in \mathbb{R},b_{g}\in {\mathbb{R}}^{F_{int}}$, which are based on the original paper (Oktay et al., 2018) but the transform and dimensions are modified for spherical data shape on icosahedrons. Instead of the 1×1×1 convolutions, channel-wise one-ring convolutions were used to linearly map those concatenated inputs, i.e., $x^{l}$ and g, on icosahedrons toward an intermediate space with ${\mathbb{R}}^{F_{int}}$ dimension. After that, the rectified linear unit (ReLU) was applied to the concatenated features before linear transformation with another one-ring convolution and sigmoid activation function for vector concatenation-based attention (Jetley et al., 2018). The gating signal forms a grid on vertices conditioned to spatial information for each level of icosahedron from skip-connection, which enables the network to combine information from multiple scales of spherical feature maps to achieve better performance. The parameters within those attention gates can be optimized with the general back-propagation-based training of neural networks.

### 2.5 Training of the model

We trained and evaluated the model with fetal cortical surfaces extracted from 55 fetuses using 5-fold cross-validation while 10% of the training samples selected were used for validation during its training. In order to increase the number of training samples, we applied three-dimensional rotational augmentation on the icosahedron. We randomly rotated input folding maps and output parcellation maps on the sphere and re-tessellated rotated maps onto the icosahedron with 40,962 vertices using the barycentric interpolation (Berrut and Trefethen, 2004). After rotational augmentations, we normalized each input folding map’s value with the *z*-score transform (Devore, 1995). For the loss function, we used the Dice loss using the Adam optimizer (Kingma and Ba, 2014) with a learning rate of 1e−3. To get the best optimal weights of the model in each fold, we monitored the Dice coefficient for the validation set and applied the learning rate reducer with a factor of 0.1 with five-epoch patience for the stagnation of validation loss. The training continued for 100 epochs and the network weights that showed the highest dice coefficient for the validation set were stored as the optimal network for each fold. The entire automatic parcellation framework was developed using Tensorflow (Dillon et al., 2017) backend, and the training and evaluation process was conducted with Nvidia RTX A5000 GPUs.

### 2.6 Evaluation of the model

We used the dice coefficient to evaluate automatic cortical surface parcellation performance, measuring the regional overlap between the predicted parcellation label and ground truth. The overall Dice coefficient is used to measure the general parcellation performance of the model according to the following formulas.


$$Dice_{\text{overall}}\left(g,p\right)=\frac{2\times \sum_{i,l}g_{i}^{l}p_{i}^{l}+\epsilon }{\left(\sum_{i,l}g_{i}^{l}+\sum_{i,l}p_{i}^{l}\right)+\epsilon }$$

The l denotes the class label and i represents the vertex. The $p_{i}^{l}$ is the one-hot encoded prediction labels at vertex *i* for class *l* from automatic parcellation models and $g_{i}^{l}$ is the ground truth label at vertex *i* for class *l* based on manual parcellation.

We also computed boundary distance for each region to compare how predicted regional boundaries are aligned with boundaries defined on ground truth. We measured surface distance over vertices from the ground truth boundaries to the boundaries of the predicted regions and used their median to represent boundary distance. For region *j* and vertex *i* on predicted boundaries defined with *k* vertices, $d_{i}^{j}$ denotes the shortest distance toward the ground truth boundary of region *j*. Likewise, we also computed their mean to represent the overall boundary distance.


$$\text{Median}\;\text{boundary}\;\text{distance}=\text{median}\left(d_{1}^{j},d_{2}^{j},d_{3}^{j},\ldots ,d_{i}^{j},d_{k}^{j}\right)$$

Additionally, we investigated the effect of parcellation performance on cortical surface measurement. For each region *j*, we computed the ground truth regional area, $\text{are}a_{GT}^{j}$, from the manual parcellation map and the predicted regional area, $\text{are}a_{\text{pred}}^{j},$ from each automatic parcellation method for each cortical parcel. Then, we computed the absolute percent error for each region and measured their mean as the overall error for each automatic parcellation method.


$$\text{Absolute}\;\text{percent}\;\text{error}\;\left(\%\right)=\left(\frac{\left|\text{are}a_{\text{pred}}^{j}-\text{are}a_{GT}^{j}\right|}{\text{are}a_{GT}^{j}}\right)*100$$

We compared the automatic parcellation performance of the proposed attention-gated spherical U-net with surface registration-based parcellation (Fischl et al., 2004), SPHARM-net (Ha and Lyu, 2022), and original spherical U-net (Zhao et al., 2019). For surface registration-based parcellation, we aligned a 29 GA template surface with predefined regional labels, constructed with a different TD fetal cohort (Serag et al., 2012; Yun et al., 2019), to the individual cortical surface using a 2D sphere-to-sphere non-rigid warping (Robbins et al., 2004). Then, we resampled the label map for the individual surfaces.

### 2.7 Statistical analysis

All statistical analyses were conducted with IBM SPSS Statistics (Version 29), IBM Corp, and MATLAB, MathWorks Inc. For the statistical comparison of the parcellation performance, all the metrics and measurements are computed subject-wisely to investigate the improvement of parcellation performance in a paired manner. We first performed paired *t*-tests on the overall Dice coefficient, median boundary distance, and mean absolute percent error of area measurements from the proposed model against the registration-based parcellation, SPHARM-net, and original spherical U-net to statistically compare their automatic parcellation performances. For the regional evaluation, we performed paired *t*-tests on the dice coefficient, boundary distance, and absolute percentage error of area measurement for each region between methods. We used the false discovery rate (FDR) control method at a *q*-value (FDR adjusted *p* value) of 0.05 to adjust for multiple comparisons (Benjamini and Hochberg, 1995).

We examined the performance according to variations of GA and gyrification. We performed linear regression analysis, Dice=GA+c, to examine the relationship between GA and parcellation performance. Furthermore, we also divided the subjects into two subgroups based on GA, early third trimester (27–33 GA) and late third trimester (33–39 GA). Even fetal brains in both subgroups are under neurodevelopment for the maturation of the brain, cortical folding structures in the late third trimester are much more similar to infant or adult brains than those in the early third trimester. We performed paired *t*-tests on the overall dice coefficients for early and late third trimester subgroups to assess how neurodevelopmental stages affect brain parcellation performance.

## 3 Results

In terms of global parcellation performance, our proposed attention-gated spherical U-net achieved an overall dice coefficient of 0.899 ± 0.020 (mean ± SD). When we compared the performance metrics across different models, it showed a significantly higher overall dice coefficient compared to the surface registration-based method (*p* < 0.001), SPHARM-net (*p* < 0.001), and the original spherical U-net (*p* = 0.002), respectively (Table 1). Also, the median boundary distance from the proposed model achieved the lowest error than other methods while showing statistical significance only compared to the surface registration-based method (*p* < 0.001) (Table 2). Lastly, the proposed model showed the lowest mean absolute percent error in surface area measurement among the parcellation methods computed across all regions showing statistical significance than the surface registration-based method (*p* < 0.001) and SPHARM-net (*p* < 0.001) (Table 3).

**Table 1:** Statistical comparisons of overall dice coefficients between the proposed model and other parcellation methods.

Parcellation method	Param	Overall dice coefficient	*t*	*p* value
Surface registration (1)	-	0.834 ± 0.036	−17.5	**<0.001**
SPHARM-net (30)	4.3 M	0.894 ± 0.020	−3.6	**<0.001**
Original spherical U-net (12)	6.7 M	0.897 ± 0.020	−3.2	**0.002**
Attention-gated spherical U-net (Proposed model)	7.6 M	**0.899 ± 0.020**	-	-

Data for dice coefficient: mean ± SD; Param: number of trainable parameters; *t*: *t*-score from the subject-wise paired *t*-test. Bold *p*-values denote statistical significance compared to the proposed model.

**Table 2:** Effect of parcellation performance on median boundary distance.

Model	Median boundary distance (mm)	*t*	*p*-value
Surface registration	3.112 ± 1.225	18.8	**<0.001**
SPHARM-net	2.484 ± 1.272	1.2	0.24
Original spherical U-net	2.483 ± 1.320	1.1	0.29
Attention-gated spherical U-net	2.471 ± 1.322	-	-

Data: mean ± SD; *t*: *t*-score from the subject-wise paired *t*-test. Bold *p*-values denote statistical significance compared to the proposed model.

**Table 3:** Effect of parcellation performance on global surface regional area measurement.

Model	Mean absolute percent error (%)	*t*	*p* value
Surface registration	16.40 ± 3.67	16.0	**<0.001**
SPHARM-net	11.15 ± 2.62	4.3	**<0.001**
Original spherical U-net	10.48 ± 2.42	0.8	0.46
Attention-gated spherical U-net	10.40 ± 2.64	-	-

Data: mean ± SD; *t*: *t*-score from the subject-wise paired *t*-test. Bold *p*-values denote statistical significance compared to the proposed model.

**Figure 3:**
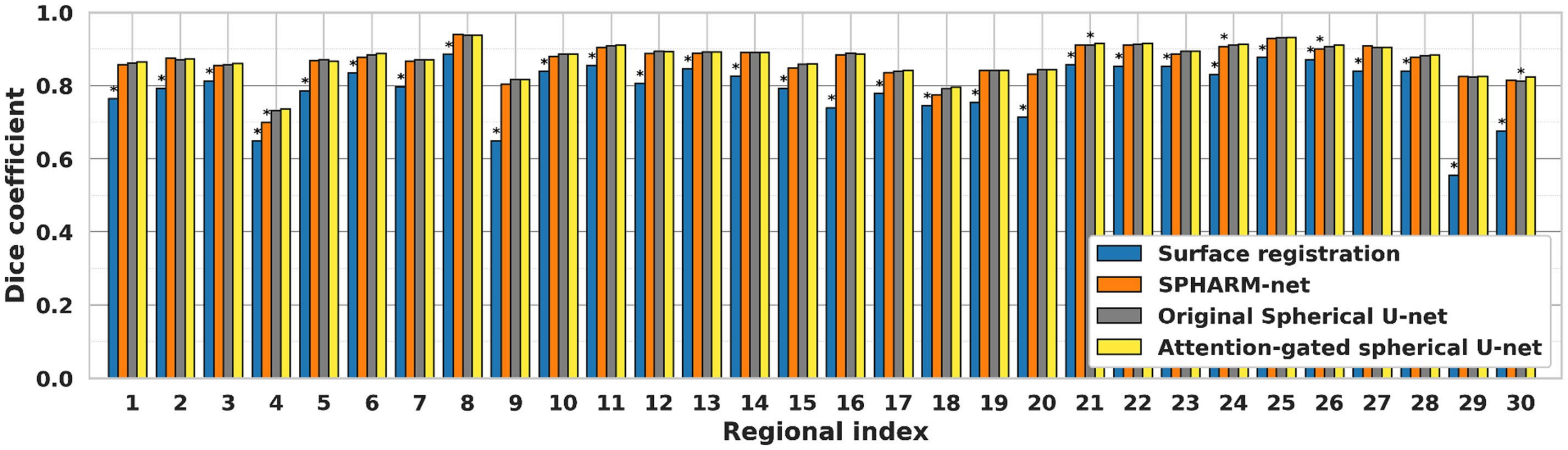
Regional dice coefficient for each automatic cortical parcellation method. ^*^Significant decrease in dice coefficient when compared to the proposed model (FDR adjusted *p* < 0.05). Label number: see Figure 1.

For regional evaluation of parcellation performance, we first compared dice coefficients for each parcellated region (Figure 3). The attention-gated spherical U-net outperformed the surface registration-based method, showing a statistically significant increase in the regional dice coefficient for most regions except for the precentral gyrus. When it comes to the comparisons against SPHARM-net and original spherical U-net, their trends of improvement were notable even though they did not retain statistical significance after FDR correction. The proposed model showed higher Dice coefficients than SPHARM-net for every region except for the insula and superior temporal gyrus. It also showed increased dice coefficients than the original spherical U-net for the caudal middle frontal gyrus, cingulate cortex, cuneus, frontal pole, inferior parietal gyrus, pars orbitalis, and transverse temporal gyrus. The improvement in the regional dice coefficient, although not statistically significant after FDR correction, indicates their trend toward the most precise parcellation performance.

**Figure 4:**
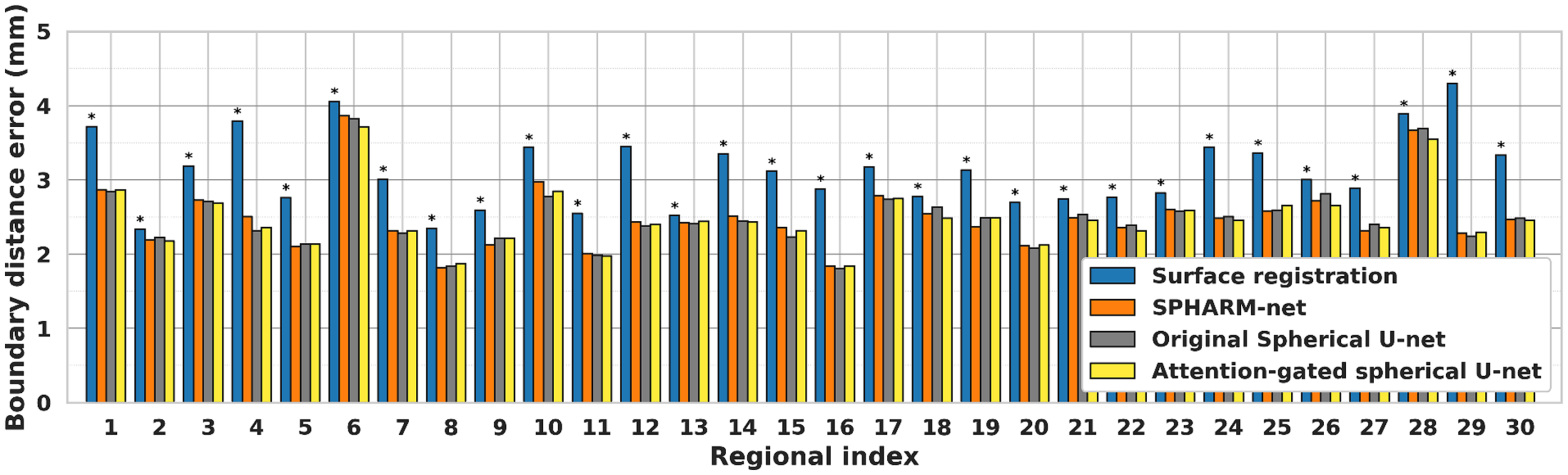
Regional median boundary distance of each automatic cortical parcellation method. ^*^Significant increase in boundary distance for each region when compared to the proposed model (FDR adjusted *p* < 0.05) Label number: see Figure 1.

Similarly, the trends of improvement are observed in both errors from boundary distance measurement and surface area measurement. The attention-gated spherical U-net showed significantly lower boundary distances than the surface registration-based method for every region except the cingulate cortex, medial orbital frontal cortex, and precuneus cortex after FDR correction. Despite not achieving statistical significance following FDR correction, the boundary distances still showed decreasing trends compared to SPHARM-net and original spherical U-net (Figure 4). In terms of regional absolute percent error of surface area measurement, the attention-gated spherical U-net showed significantly lower errors from the surface registration-based method for every region except medial orbital frontal cortex, pars opercularis, and pars orbitalis after FDR correction. It also showed statistically lower errors in the frontal pole, paracentral lobule, and precuneus cortex compared to SPHARM-net. However, the attention-gated spherical U-net did not achieve statistical significance against the original spherical U-net even though it showed decreasing trends (Table 4).

**Table 4:** Effect of parcellation performance on regional surface regional area measurement.

Regional absolute percent error (%)				
Label index	Surface registration	SPHARM-net	Original spherical U-net	Attention-gated spherical U-net
1	16.7 + 13.5*	13.7 ± 12.3	12.5 ± 11.4	12.0 ± 10.8
2	13.6 ± 11.2*	10.1 ± 9.0	9.6 ± 7.9	8.9 ± 7.7
3	24.0 ± 21.4*	10.9 ± 10.5	12.3 ± 12.7	11.3 ± 12.0
4	64.0 ± 67.8*	31.7 ± 27.3*	24.1 ± 21.3	24.2 ± 19.9
5	12.2 ± 10.1*	10.7 ± 9.8	9.6 ± 8.1	9.6 ± 10.5
6	13.3 ± 11.2*	10.6 ± 9.3	10.2 ± 8.8	10.9 ± 9.5
7	14.8 ± 10.7*	10.3 ± 9.8	9.9 ± 8.3	10.4 ± 9.6
8	12.9 ± 11.3*	4.6 ± 3.5	3.6 ± 2.8	3.9 ± 2.7
9	25.6 ± 14.9*	16.2 ± 12.1	15.2 ± 12.1	15.2 ± 13.6
10	13.1 ± 12.6*	10.7 ± 8.8	10.9 ± 10.3	10.7 ± 10.5
11	11.5 ± 7.6*	8.0 ± 7.1	6.9 ± 5.6	6.7 ± 5.8
12	11.1 ± 10.2*	9.9 ± 8.9	8.2 ± 7.7	8.6 ± 8.0
13	**11.3 ± 9.6**	10.1 ± 8.2	9.9 ± 10.1	10.1 ± 9.7
14	14.8 ± 10.7*	8.5 ± 8.0	8.3 ± 6.4	8.7 ± 6.6
15	16.8 ± 13.8*	15.2 ± 12.4*	13.6 ± 9.7	12.4 ± 10.2
16	16.5 ± 12.5*	10.0 ± 8.5	9.7 ± 7.6	10.0 ± 7.9
17	**13.1 ± 10.8**	13.8 ± 15.3	14.6 ± 13.9	14.2 ± 13.3
18	**17.5 ± 14.3**	15.1 ± 14.3	18.1 ± 17.3	17.6 ± 15.8
19	22.8 ± 12.5*	12.2 ± 8.9	12.3 ± 10.2	12.9 ± 11.2
20	16.0 ± 16.0*	15.1 ± 13.8	12.5 ± 12.7	12.4 ± 12.1
21	6.4 ± 6.4*	5.9 ± 5.5	5.2 ± 4.4	4.9 ± 4.0
22	8.5 ± 8.5*	5.4 ± 3.8	5.3 ± 3.5	5.4 ± 3.9
23	13.6 ± 11.0*	9.2 ± 7.3*	7.0 ± 5.9	6.8 ± 5.8
24	13.0 ± 8.0*	6.9 ± 6.6	7.1 ± 5.6	6.5 ± 5.7
25	8.5 ± 6.6*	5.9 ± 4.7	5.1 ± 4.7	5.5 ± 4.8
26	10.9 ± 10.4*	8.8 ± 9.5	7.8 ± 7.3	7.5 ± 5.9
27	13.0 ± 9.7*	6.8 ± 5.9	7.1 ± 5.0	6.9 ± 5.1
28	16.0 ± 16.5*	9.5 ± 8.5	8.7 ± 6.5	8.8 ± 7.9
29	21.3 ± 15.4*	16.0 ± 14.1	16.5 ± 14.6	16.7 ± 14.1
30	25.3 ± 23.7*	19.6 ± 16.0	19.6 ± 16.9	19.5 ± 17.0

Data: mean ± SD; ^*^Significant difference compared to the attention-gated spherical U-net after multiple-comparison correction with FDR at *q* = 0.05; Label number: see Figure 1. Bold *p*-values denote statistical significance compared to the proposed model.

**Figure 5:**
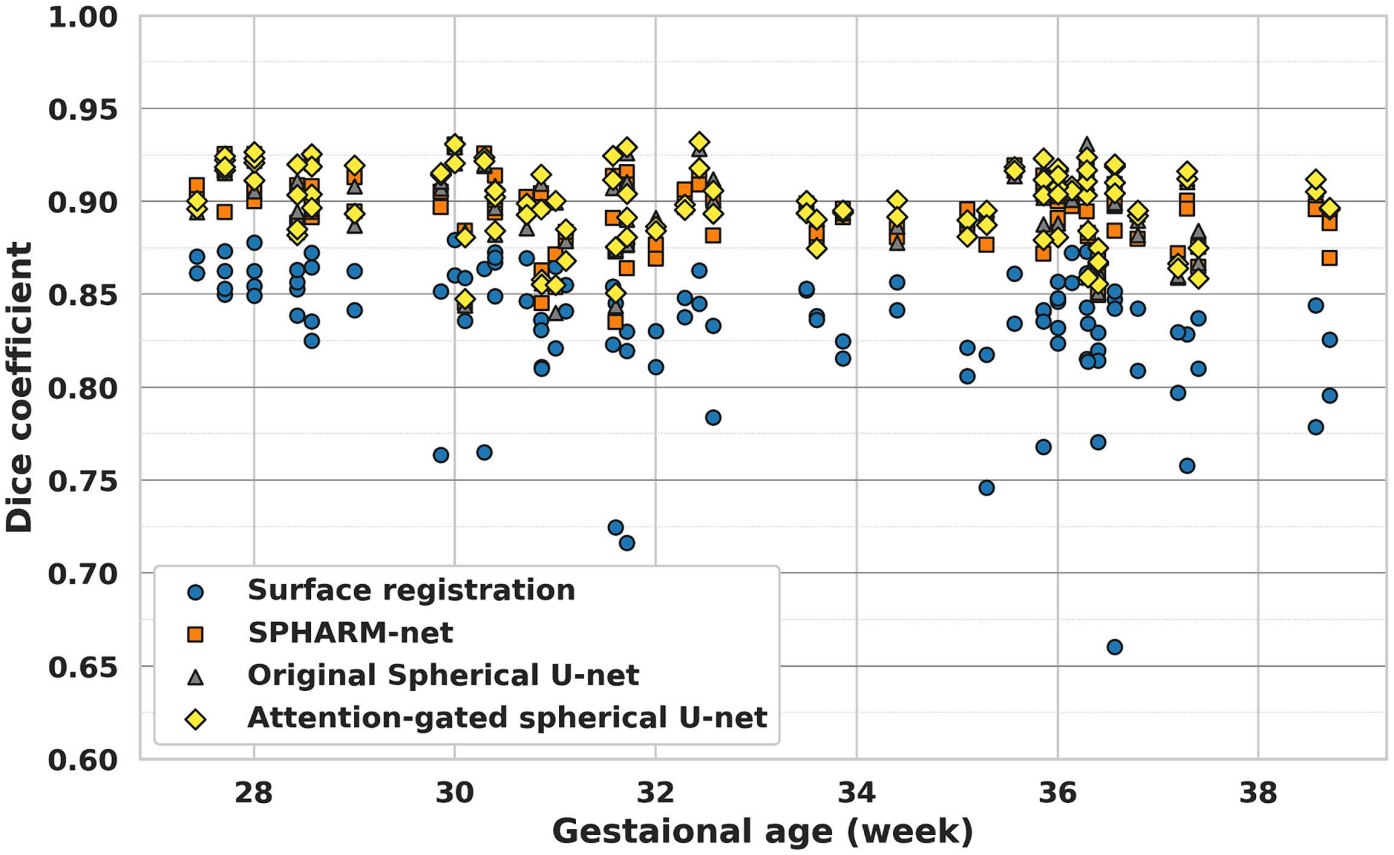
Cortical parcellation performance of each method along with GA.

**Figure 6:**
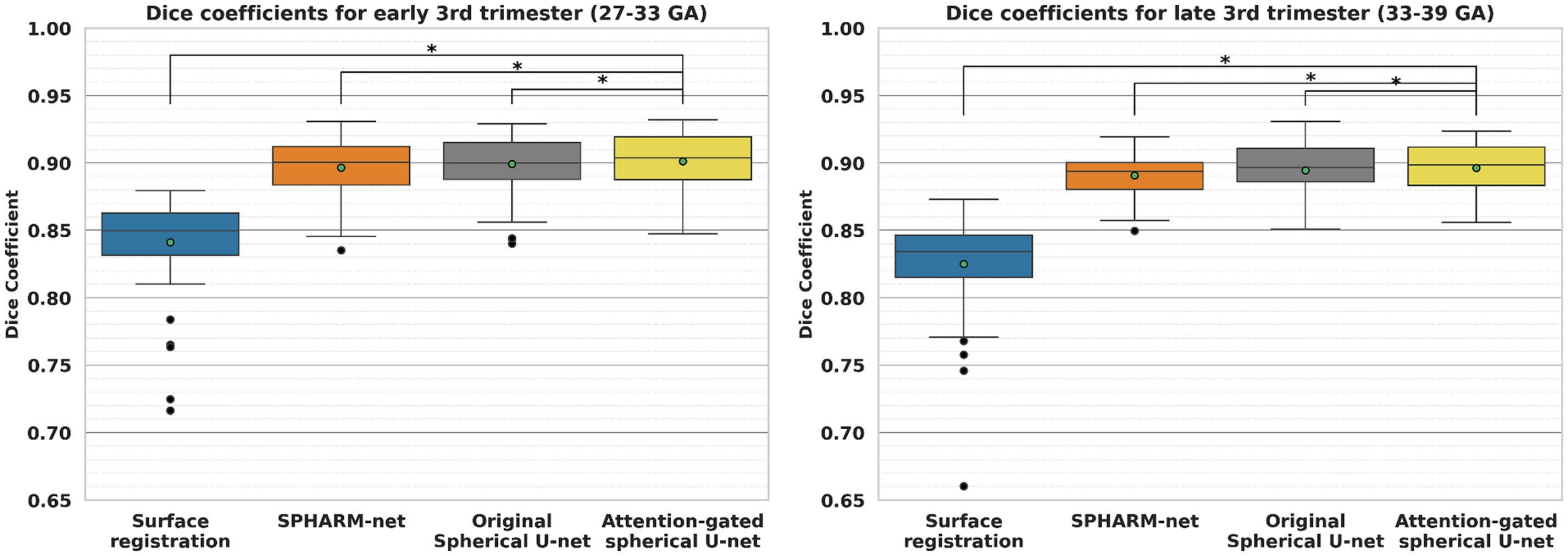
Comparison of cortical parcellation performance within subgroups, early third trimester (27–33 GA) and late third trimester (33–39 GA). ^*^Significant decrease in dice coefficient when compared to the proposed model (*p* < 0.05).

When it comes to the effect of GA on parcellation, the overall dice coefficient from the proposed model was not statistically associated with GA $\left(p=0.055\right)$, while the surface registration-based method (p<0.001) and SPHARM-net $\left(p=0.008\right)$ showed a statistically significant negative correlation (Table 5; Figure 5). Furthermore, from the subgroup analysis dividing the subjects into two subgroups based on GA: early third trimester (27–33 GA) and late third trimester (33–39 GA), the proposed model showed the highest dice coefficients in both subgroups maintaining statistical significance (*p* < 0.05). It highlights its robustness across varying stages of brain development (Figure 6).

**Table 5:** Result of linear regression analysis between GA and overall dice coefficient.

Model	$R^{2}$	Standardized coefficients beta for GA	*t*	*p* value
Surface registration	0.11	−0.33	−3.66	**<0.001**
SPHARM-net	0.06	−0.25	−2.71	**0.008**
Original spherical U-net	0.03	−0.17	−1.76	0.082
Attention-gated spherical U-net	0.03	−0.19	−1.95	0.055

Bold *p*-values denote statistical significance compared to the proposed model.

## 4 Discussion

In this study, we proposed the attention-gated spherical U-net for cortical surface parcellation in the fetal brain. The proposed model outperformed the surface registration-based method, SPHARM-net, and the original spherical U-net. The proposed model exhibited robustness across different GAs, showing no statistically significant association with overall Dice coefficients. Subgroup analysis further confirmed its high performance and robustness across early and late third trimesters of brain development.

### 4.1 Cortical surface parcellation performance in the fetal brain

**Figure 7:**
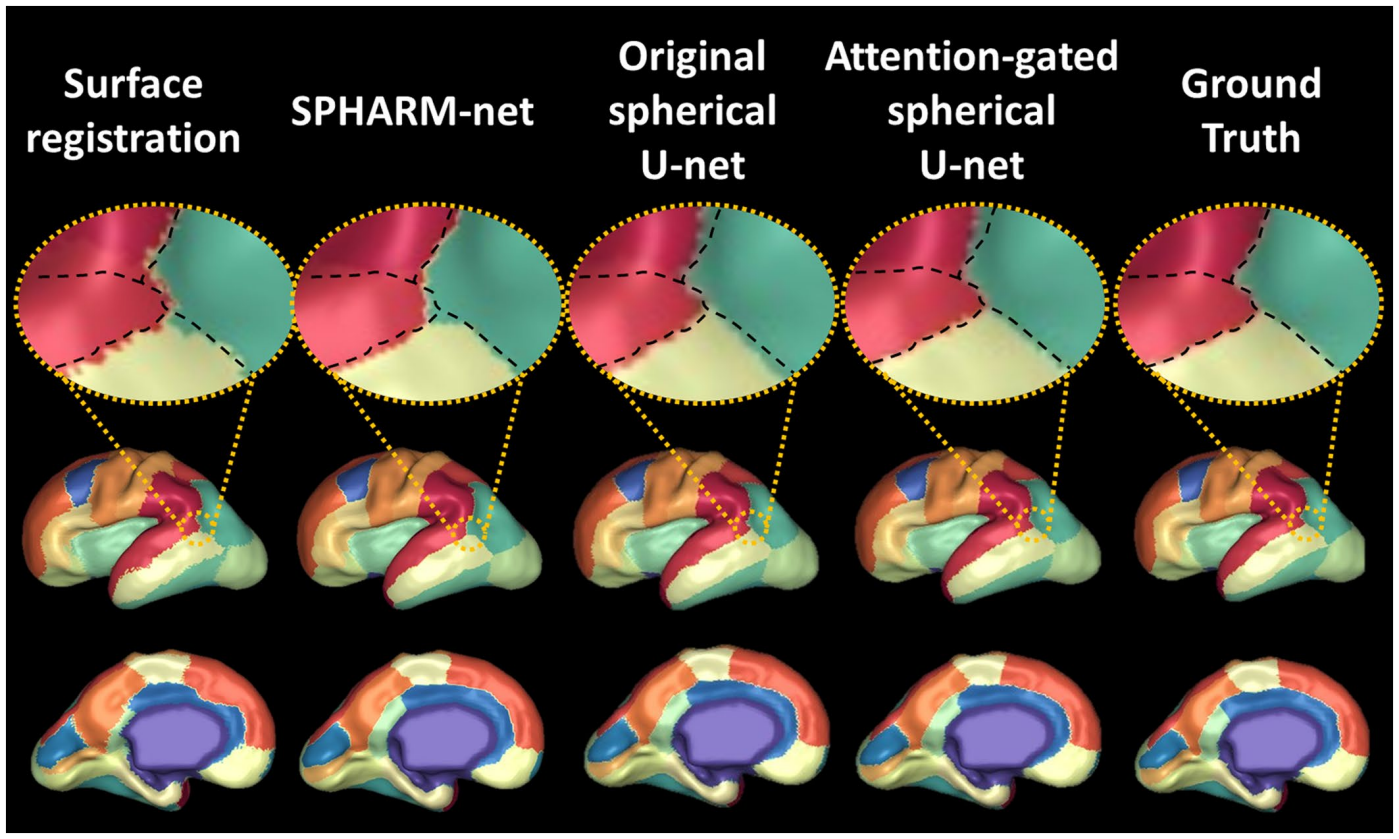
Visualization of automatic cortical surface parcellation outputs in the fetal brain. Comparison of predicted cortical parcellation label map on a fetal brain with 28 GA and its ground truth. On the top row, the black dotted line indicates the boundaries between four regions (scarlet, red, green, lime) from ground truth. The attention-gated spherical U-net shows smoother boundaries mostly aligned with the ground truth.

Previous cortical surface parcellation methods are not sufficient for parcellation in fetal brains which have small size, limited gyrification, and large temporal variation since those methods rely on the presence of well-defined gyral and sulcal patterns. These differences require the parcellation model to capture information both adaptively and sensitively. The attention mechanism has emerged as a pivotal component in deep learning models, particularly in tasks that require discerning intricate patterns and relationships within data (Oktay et al., 2018; Schlemper et al., 2019). In the context of cortical surface parcellation, the attention mechanism plays a crucial role in instructing the model to focus on specific patterns on the cortical feature maps, which are relevant to infer the parcellation outputs (Jetley et al., 2018; Liu et al., 2022), thereby it contributes to improving the accuracy and precision of the automatic parcellation in fetal brains. The statistically significant improvements in overall dice coefficients (Table 1), as compared to previous methods, demonstrate the effect of attention mechanisms on fetal cortical parcellation. By focusing on relevant features and suppressing irrelevant ones, the attention mechanism allowed the model to capture fine-grained boundaries more accurately, which leads to the most similar boundaries of ground truth (Figure 7) and lowest boundary distances among the automatic parcellation approaches both globally and locally (Table 2; Figure 4). This is particularly important in cortical surface parcellation in fetal brains, where the distinction between different gyral regions can be subtle.

Furthermore, the attention mechanism aids in contextual understanding (Zhang et al., 2018). In the complex landscape of the brain, understanding the contextual information such as global and local folding patterns of the cortical surface is important to infer regional labels on the cortical surface. The attention mechanism allows the model to weigh the importance of different regions based on their context, leading to more accurate parcellation (Chen et al., 2018). The region-wise evaluation further reinforces the superiority of the attention-gated spherical U-net since each cortical region has a different rate of growth and gyrification. In the regions where cortical folding formed in the early developmental period, such as the precentral gyrus, postcentral gyrus, and insula, all of the parcellation methods showed stable performances. On the other hand, the proposed model showed greater performance in the cortical regions with late gyrification, such as the caudal middle frontal gyrus, cuneus, inferior parietal gyrus, par orbitalis, and transverse temporal gyrus (Figure 3). Not only did it outperform the surface registration-based method, but it also showed significant improvements against other deep learning models like SPHARM-net and the original spherical U-net. This suggests that the attention mechanism is not just a supplementary feature but a core component that substantially enhances the model’s performance.

Lastly, the improvement of the parcellation performance with the proposed model was not limited to the evaluation via dice coefficients. The proposed model showed the lowest error in the actual cortical surface measurements both globally and regionally (Tables 3, 4). This result emphasizes the effectiveness of the attention mechanism for automatic cortical surface parcellation, which eventually leads to precise regional analysis of the fetal brain.

### 4.2 Parcellation performance along GA

Analyzing parcellation performance along GA provides valuable insights into the model’s adaptability and accuracy across different developmental stages. The fetal brains in the third trimester are under rapid growth of the cerebral cortex with gyrification, forming foldings on the surfaces. It means that the region belonging to the same parcellation label could have large temporal variations on its folding feature maps along with GA. For the precise parcellation of fetal brains across a wide range of GA, the model is required to find peculiar patterns composing each cortical region and focus on them, such as its sulcus/gyrus and their relative relationship, despite the large temporal variation in fetal brains.

The result shows the usefulness of the attention-gated spherical U-net for fetal cortical surface parcellation across a wide range of GA (Table 5; Figure 5). The surface registration-based method showed lower dice coefficients for all GAs and a bigger variation in subjects with higher GA, which led negative correlation between GA and dice coefficients. The SPHARM-net showed slightly lower performance around 31 weeks and decreased dice coefficients with GA. Our attention-gated spherical U-net was less influenced by the variations in brain development stages, suggesting that it could be a more reliable tool for fetal studies across different GA. Furthermore, the subgroup analysis dividing subjects into early (27–33 GA) and late third trimester (33–39 GA) subgroups further emphasizes the proposed model’s capability to adapt to the dynamic nature of fetal brain development during this period showing the highest overall Dice coefficients within both subgroups (Figure 6).

### 4.3 Limitations

The proposed attention-gated spherical U-net showed improved performance in cortical parcellation of the fetal brain, there are some limitations to be addressed in future studies. First, the fetal MRIs used in this study were sourced from a single center. This may introduce biases related to specific imaging protocols, equipment, and patient demographics. Multi-center studies are essential to validate the generalizability of our findings across different settings and populations. Secondly, the number of subjects included in this study is relatively smaller than other cortical brain parcellation studies due to the rarity of fetal MRIs. For future research, a collaborative multi-center approach with a larger cohort is recommended to gather a diverse set of fetal MRI data, ensuring broader generalizability. Lastly, constructing a comprehensive dataset including possible variations by maternal health, socioeconomic status, racial and ethnic background, and other clinical factors will enhance the model’s adaptability and accuracy in reflecting real-world variability in fetal brain development.

## 5 Conclusion

This study introduced the attention-gated spherical U-net for automatic cortical surface parcellation of the fetal brain, showing its improved performance over the conventional surface registration-based method and other previously developed deep learning models. The proposed model could work as a valuable tool for precise regional analyses in fetuses that help understand brain development and neurodevelopment disorders. Furthermore, it could increase sensitivity to detect abnormalities in specific regions, which leads to the potential for early detection of neurodevelopmental disorders in the future.

## Data availability statement

The raw data supporting the conclusions of this article will be made available by the authors, without undue reservation.

## Ethics statement

The studies involving humans were approved by Institutional Review Board at Boston Children’s Hospital. The studies were conducted in accordance with the local legislation and institutional requirements. Written informed consent for participation in this study was provided by the participants’ legal guardians/next of kin. Written informed consent was obtained from the individual(s) for the publication of any potentially identifiable images or data included in this article.

## Author contributions

SY: Writing – original draft, Writing – review & editing, Conceptualization, Data curation, Formal analysis, Methodology, Software, Visualization. AL: Data curation, Writing – review & editing. VC: Data curation, Writing – review & editing. HY: Writing – review & editing. EY: Data curation, Writing – review & editing. PG: Writing – review & editing, Investigation, Resources, Supervision. KI: Writing – review & editing, Conceptualization, Investigation, Resources, Supervision.

## Footnotes

^1^ https://github.com/FNNDSC/fetal-brain-segmentation

^2^ https://mcin.ca/technology/civet/
